# Literature Based Drug Interaction Prediction with Clinical Assessment Using Electronic Medical Records: Novel Myopathy Associated Drug Interactions

**DOI:** 10.1371/journal.pcbi.1002614

**Published:** 2012-08-09

**Authors:** Jon D. Duke, Xu Han, Zhiping Wang, Abhinita Subhadarshini, Shreyas D. Karnik, Xiaochun Li, Stephen D. Hall, Yan Jin, J. Thomas Callaghan, Marcus J. Overhage, David A. Flockhart, R. Matthew Strother, Sara K. Quinney, Lang Li

**Affiliations:** 1 Regenstrief Institute, Indianapolis, Indiana, United States of America; 2 Department of Pharmacology and Toxicology, School of Medicine, Indiana University, Indianapolis, Indiana, United States of America; 3 Division of Clinical Pharmacology, Department of Medicine, School of Medicine, Indiana University, Indianapolis, Indiana, United States of America; 4 Center for Computational Biology and Bioinformatics; School of Medicine, Indiana University, Indianapolis, Indiana, United States of America; 5 Department of Medical and Molecular Genetics, School of Medicine, Indiana University, Indianapolis, Indiana, United States of America; 6 Department of Biostatistics, School of Medicine, Indiana University, Indianapolis, Indiana, United States of America; 7 Eli Lilly Inc., Indianapolis, Indiana, United States of America; 8 Siemens, Malvern, Pennsylvania, United States of America; 9 Indiana Institute of Personalized Medicine, School of Medicine, Indianapolis, Indiana, United States of America; 10 Division of Hematology and Oncology, Department of Medicine, Indiana University, Indianapolis, Indiana, United States of America; 11 Department of Obstetrics and Gynecology, School of Medicine, Indiana University, Indianapolis, Indiana, United States of America; Stanford University, United States of America

## Abstract

Drug-drug interactions (DDIs) are a common cause of adverse drug events. In this paper, we combined a literature discovery approach with analysis of a large electronic medical record database method to predict and evaluate novel DDIs. We predicted an initial set of 13197 potential DDIs based on substrates and inhibitors of cytochrome P450 (CYP) metabolism enzymes identified from published *in vitro* pharmacology experiments. Using a clinical repository of over 800,000 patients, we narrowed this theoretical set of DDIs to 3670 drug pairs actually taken by patients. Finally, we sought to identify novel combinations that synergistically increased the risk of myopathy. Five pairs were identified with their p-values less than 1E-06: loratadine and simvastatin (relative risk or RR = 1.69); loratadine and alprazolam (RR = 1.86); loratadine and duloxetine (RR = 1.94); loratadine and ropinirole (RR = 3.21); and promethazine and tegaserod (RR = 3.00). When taken together, each drug pair showed a significantly increased risk of myopathy when compared to the expected additive myopathy risk from taking either of the drugs alone. Based on additional literature data on *in vitro* drug metabolism and inhibition potency, loratadine and simvastatin and tegaserod and promethazine were predicted to have a strong DDI through the CYP3A4 and CYP2D6 enzymes, respectively. This new translational biomedical informatics approach supports not only detection of new clinically significant DDI signals, but also evaluation of their potential molecular mechanisms.

## Author Summary

Drug-drug interactions are a common cause of adverse drug events. In this paper, we developed an automated search algorithm which can predict new drug interactions based on published literature. Using a large electronic medical record database, we then analyzed the correlation between concurrent use of these potentially interacting drugs and the incidence of myopathy as an adverse drug event. Myopathy comprises a range of musculoskeletal conditions including muscle pain, weakness, and tissue breakdown (rhabdomyolysis). Our statistical analysis identified 5 drug interaction pairs: (loratadine, simvastatin), (loratadine, alprazolam), (loratadine, duloxetine), (loratadine, ropinirole), and (promethazine, tegaserod). When taken together, each drug pair showed a significantly increased risk of myopathy when compared to the expected additive myopathy risk from taking either of the drugs alone. Further investigation suggests that two major drug metabolism proteins, CYP2D6 and CYP3A4, are involved with these five drug pairs' interactions. Overall, our method is robust in that it can incorporate all published literature, all FDA approved drugs, and very large clinical datasets to generate predictions of clinically significant interactions. The interactions can then be further validated in future cell-based experiments and/or clinical studies.

## Introduction

Drug-drug interactions (DDIs) are a major cause of morbidity and mortality and lead to increased health care costs [1]–[3]. DDIs are responsible for nearly 3% of all hospital admissions [4] and 4.8% of admissions in the elderly [1]. And with new drugs entering the market at a rapid pace (35 novel drugs approved by the FDA in 2011), identification of new clinically significant drug interactions is essential. DDIs are also a common cause of medical errors, representing 3% to 5% of all inpatient medication errors [5]. These numbers may actually underestimate the true public health burden of drug interactions as they reflect only well-established DDIs.

Several methodological approaches are currently used to identify and characterize new DDIs. *In vitro* pharmacology experiments use intact cells (e.g. hepatocytes), microsomal protein fractions, or recombinant systems to investigate drug interaction mechanisms. The FDA provides comprehensive recommendations for *in vitro* study designs, including recommended probe substrates and inhibitors for various metabolism enzymes and transporters [6]. The drug interaction mechanisms and parameters obtained from these *in vitro* experiments can be extrapolated to predict *in vivo* changes in drug exposure. For example, a physiologically based pharmacokinetics model was developed to predict the clinical effect of mechanism based inhibition of CYP3A by clarithromycin from *in vitro* data [7]. However, *in vitro* experiments alone often cannot determine whether a given drug interaction will affect drug efficacy or lead to a clinically significant adverse drug reaction (ADR).

*In vivo* clinical pharmacology studies utilize either randomized or cross-over designs to evaluate the effect on an interaction on drug exposure. Drug exposure change serves as a biomarker for the direct DDI effect, though drug exposure change may or may not lead to clinically significant change in efficacy or ADRs. The FDA provides well-documented guidance for conducting *in vivo* clinical pharmacology DDI studies [6]. If well-established probe substrates and inhibitors are used, involvement of specific drug metabolism or transport pathway can be demonstrated by *in vivo* clinical studies. For example, using selective probe substrates of OATPs (pravastatin) and CYP3A (midazolam) and probe inhibitors of OATPs (rifampicin) and CYP3A (itraconazole), it was shown that hepatic uptake via OATPs made the dominant contribution to the hepatic clearance of atorvastatin in an *in vivo* clinical PK study [8]. However, due to overlap in substrate selectivity, an *in vivo* DDI study alone will often not provide mechanistic insight into the DDI.

Finally, *in populo* pharmacoepidemiology studies use a population-based approach to investigate the effect of a DDI on drug efficacy and ADRs. For example, the interactions between warfarin and several antibiotics were evaluated for increased risk of gastrointestinal bleeding and hospitalization in a series of case-control and case-crossover studies using US Medicaid data [9]. Indeed, epidemiological studies using large clinical datasets can identify potentially interacting drugs within a population, but these studies alone are insufficient to characterize pharmacologic mechanisms or patient-level physiologic effects.

The aforementioned *in vitro*, *in vivo*, and *in populo* research methods are complementary in characterizing new drug-drug interactions. Yet these methods are all limited by their relatively small scale. Such studies usually focus on a few drug pairs for one or a limited number of metabolizing enzymes or transporters a time. Performing large scale screening for novel drug interactions requires higher throughput strategies. Literature mining and data mining have become powerful tools for knowledge discovery in biomedical informatics, and are particularly useful for hypothesis generation. A recent notable example in clinical pharmacology is the successful detection of novel DDIs through mining of the FDA's Adverse Event Reporting System [10]. In this study, pravastatin and paroxetine were found to have a synergistic effect on increasing blood glucose. This finding was validated in three large electronic medical record (EMR) databases. While a ground-breaking success, this approach provides little evidence regarding the mechanism of the interaction.

In this paper, we present a novel approach using literature mining for screening of potential DDIs based on mechanistic properties, followed by EMR-based validation to identify those interactions that are clinically significant. We focus on clinically and statistically significant DDIs that increase the risk of myopathy.

## Results

### Literature Mined CYP Enzyme Substrates and Inhibitors

**Figure 1:**
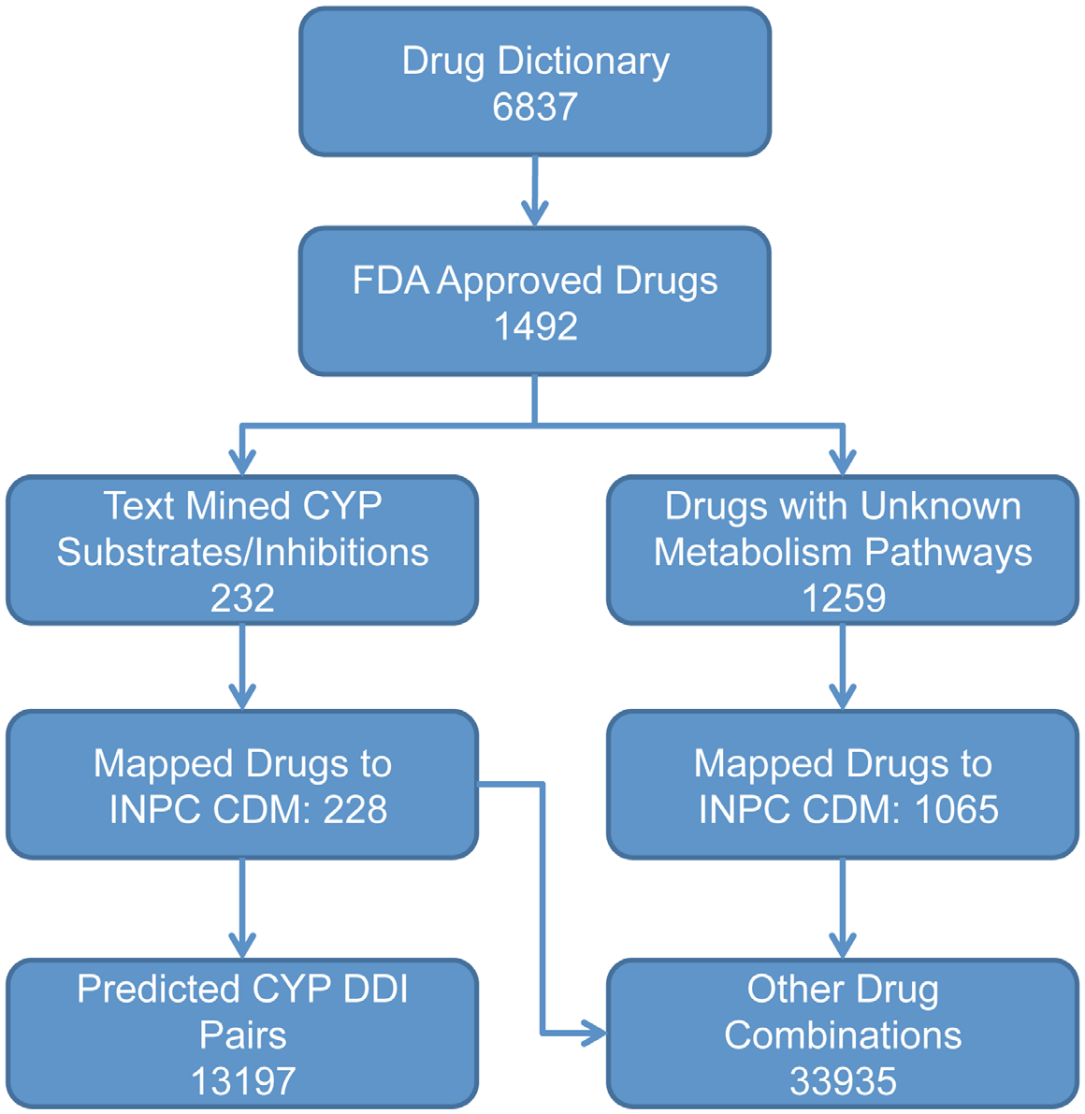
Drug names and drug interaction pairs filtering and mapping flow chart.

Our initial drug dictionary consisted of 6937 drugs. Of these, 1492 drugs were validated as FDA approved drugs (Figure 1). Among these 1492 drugs, our text mining approach identified 232 drugs, as either CYP substrates or inhibitors (Table S1). Recall rate (i.e. the proportion of true positives identified by the text mining method among all the true positives) and accuracy (i.e. the proportion of true positives among the text mined results) were used to evaluate the text mining performance. The recall rate of this text mining analysis was 0.97, with the information retrieval (IR) step being rate-limiting. In the information extraction (IE) step, the two initial curators agreed on 78% of cases. The third curator was able to establish DDI relevance and extract information in the 22% of cases which were in disagreement. The third curator also confirmed 100% accuracy among 20% of randomly chosen abstracts that the first two curators had agreed upon. Therefore, the accuracy of our text mining analysis reached 100%.

These drugs' metabolism and inhibition enzymes were experimentally determined by probe substrates and inhibitors recommended by the FDA Drug-Drug Interaction guidelines. Their categorizations are reported in Table S1. Out of the 149 CYP substrates identified, 102 (68%), were substrates of CYP3A4/5. This was consistent with the literature that about half of the drugs on the market which undergo metabolism are metabolized by CYP3A [11]. A total of 59 drugs were found to undergo metabolism by more than one CYP enzyme. We also identified 123 CYP inhibitors, with CYP3A4/5, CYP2D6, CYP2C9, CYP1A2, and CYP2C19 having comparable numbers of inhibitors, (48, 39, 39, 39, 31 respectively). Fewer inhibitors were identified for other enzymes. Fifty inhibitors were found to inhibit more than one enzyme.

### Predicted Metabolism Based DDIs and Their Clinical Pharmacokinetics DDI Validation

**Figure 2:**
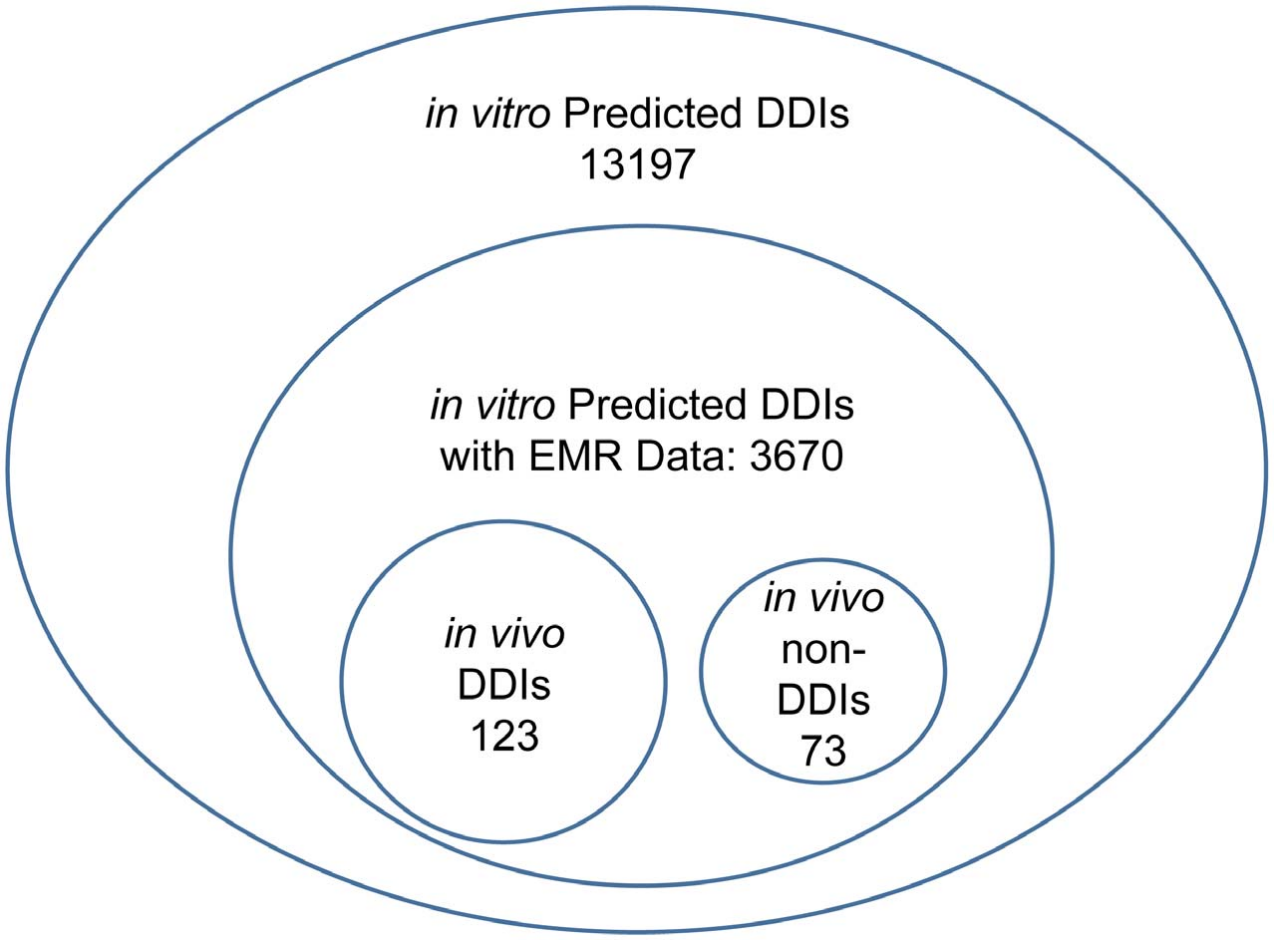
The van-diagram of predicted DDIs, DDIs with EMR data, and DDIs tested *in vivo*. The predicted DDIs were from the literature mining. DDIs with EMR data mean DDIs with non-zero frequency among the co-medication data in the EMR. *in vivo* DDIs mean that DDIs were shown changing substrate concentration significantly (p<0.05 or fold-change>2); and *in vivo* non DDIs mean that DDIs were not shown changing substrate concentration significantly.

Among 232 drugs with known metabolism and/or inhibition enzyme information (Figure 1), 13,197 drug interaction pairs were predicted based on their pertinent CYP enzymes (Figure 2). Among these 13,197 predicted DDIs, 3670 DDI pairs were prescribed as co-medications in actual patients within the Common Data Model (CDM) dataset. In other words, these 3670 predicted DDI pairs may have potential real-world clinical implication.

Among those 3670 predicted DDI pairs from *in vitro* studies, text mining identified 196 pairs with published clinical drug-drug interaction study results. These *in vivo* studies tested whether a substrate drug's exposure (i.e. systemic drug concentration) was increased when co-administrating with an inhibitor. The recall rate of this text mining analysis was 0.94. The accuracy of this text mining analysis reached 100%, after manual IE from two curators and validation from the third. Among these 196 *in vivo* validated DDI pairs, 123 of them were found to have significant DDIs (Figure 2), i.e. drug exposure increased significantly (P<0.05), and it increased by more than 2 fold. The additional 73 pairs were considered not to be clinically significant DDI's.

### EMR Data Description and Demographic Variable Effect on Myopathy

In our CDM dataset, there were medication records on 817,059 patients. Among these patients, 59,572 (7.2%) experienced myopathy events (Table 1). Two major subcategories of myopathy: myalgia and myositis/muscle weakness accounted for more than 95% of the cases. There were 53 rhabdomyolysis cases. In the cohort of individuals suffering a myopathy event, the average age was 40.2 year (SD = 23 years); 59.1% were female, and the average medication frequency was 3.8 (SD = 2.5). However, 65.8% of the race data were missing. In our initial data analysis, we found that females had higher myopathy risk than males (8.6% vs 5.4%, p<2e-16, Table 2); and each one year increase in age was associated with 0.15% higher myopathy risk (p<2e-16). These results were consistent with the literature [12].

**Table 1:** Demographic tables.

Variables	Characteristics			
Myopathy		Myopathy Concept ID	Myopathy Concept Name	Frequency
Yes	59,572 (7.2%)	446370	Antilipemic and antiarteriosclerotic drugs causing adverse effects in therapeutic use	206
No	769,333 (92.8%)	4262118	Other myopathies	7
		80800	Polymyositis	372
		73001	Myositis	53
		84675	Myalgia and myositis	48877
		4217978	Myalgia and myositis, unspecified	185
		439142	Myoglobinuria	52
		4147768	Myopathy, unspecified	1
		4345578	Rhabdomyolysis	52
		4248141	Rhabdomyolysis	1
		79908	Muscle weakness	12720
		4218609	Muscle weakness (generalized)	22
**Age (year)**	40.2+/−23.0 (11,846 missing)			
**Sex**	Female	489,669 (59.1%)		
	Male	327,390 (39.5%)		
	missing	11,846 (1.4%)		
**Medication Frequency**	3.8+/−2.5			
**Race**	White	185,675	22.4%	
	Black	65,484	7.9%	
	Asian	1,741	0.2%	
	Hispanic	30,670	3.7%	
	Native American	61	0.0073%	
	Missing	545,277	65.8%	

Note: some of the myopathy Concept ID categories overlapped.

**Table 2:** Demographic variable effect on myopathy.

Variables	Effect		
**Sex**	Male	0.054 (0.00045)	
	Female	0.086 (0.00067)	
	OR	1.64+/−0.0039	p-value<2e-16
**Age** (year)		1.0015+/−0.000012	p-value<2e-16

### Global Test of DDI Effects on Myopathy

**Figure 3:**
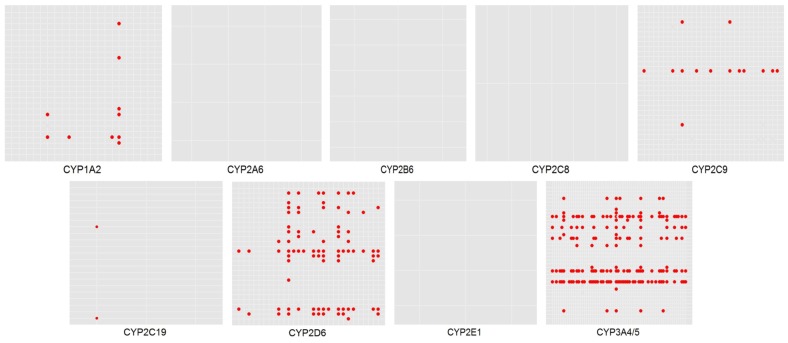
DDI enrichment plots among 9 CYP enzymes. Both x- and y-axis represent different drug names from a DDI pair. A red-dot highlights a DDI pair showing a strong association with myopathy risk (p<0.0000136, odds ratio>1).

The 3670 DDI pairs identified in the CDM database were tested using the additive model, i.e. whether an inhibitor would increase the myopathy risk of the substrate compared to the substrate alone. Both age and sex were justified in the logistic regression. The p-value threshold was chosen as 0.05/3670 = 0.0000136 after Bonferroni justification, with OR greater than 1. There were 124 and 287 significant DDI pairs for CYP2D6 and CYP3A4/5 enzymes, respectively (Figure 3 and Table S2). The other enzymes had fewer significant DDI pairs. Pathway enrichment analysis suggested similar results, i.e. CYP2D6 and CYP3A4/5 enzymes had more significant DDI pairs than the other enzymes, p = 8E-8 and 4E-2 respectively. Although this DDI analysis was confounded by the other co-medication variables, it was indeed a global description of DDI effects from various CYP enzymes. This global analysis provided us a picture of the metabolism enzymes that were most important in understanding the increased myopathy risk associated with DDIs.

### Synergistic DDI Effects on Myopathy

In order to remove the effect of myopathy risk of the inhibitor itself, a synergistic DDI test was conducted to determine whether substrate and inhibitor together have higher risk than the combined additive risk when the substrate or inhibitor is taken alone. Both age and sex were justified as covariates. DDI pairs were removed if either one of the drugs was prescribed to treat symptoms of myopathy. We set the significance threshold as p = 0.0000136, as justified the multiple primary hypotheses on 3670 predicted DDI pairs. Table 3 presents the five significant synergistic DDI pairs: (loratadine, simvastatin), (loratadine, alprazolam), (loratadine, duloxetine), (loratadine, ropinirole), and (promethazine, tegaserod). Their relative risks were (1.69, 1.86, 1.94, 3.21, 3.00) respectively, the p-values were (2.03E-07, 2.44E-08, 5.60E-07, 2.60E-07, 2.60E-07, 8.22E-07) respectively, and their associated enzymes were primarily CYP3A4/5 and CYP2D6.

**Table 3:** DDI-Myopathy analysis adjusted for age and sex.

drug 1	drug 2	enzymes	Risk1	Risk2	Risk12	Risk Ratio	p-value	sample size (m1/n1, m2/n2, m12/n12)
**Loratadine**	**Simvastatin**	CYP3A4	0.022	0.033	0.093	1.69	2.03E-07	(1264/44245, 4197/102345, 137/1223)
**Loratadine**	**Alprazolam**	CYP3A4	0.022	0.029	0.095	1.86	2.44E-08	(1257/43341, 2251/52341, 176/1448)
**Loratadine**	**Duloxetine**	CYP2D6	0.020	0.047	0.130	1.94	5.60E-07	(1220/43552, 1385/23470, 90/631)
**loratadine**	**ropinirole**	CYP2D6	0.020	0.018	0.122	3.21	2.60E-07	(1218/43491, 164/6531, 17/123)
**promethazine**	**tegaserod**	CYP2D6	0.011	0.020	0.093	3.00	8.22E-07	(1332/78334, 109/3745, 23/224)

Note: Risk1 and risk2 are myopathy risks for drug 1 and drug 2 respectively. The risk-ratio is calculated as risk12/(risk1+risk2). The p-value is calculated from a multivariate logistic regression, in which age and sex were included. (n1, n2, n12) are sample sizes for drug exposure groups of drug 1 alone, drug 2 alone, and both drugs, respectively; and (m1, m2, m12) are myopathy frequencies for drug exposure groups of drug 1 alone, drug 2 alone, and both drugs, respectively.

Additional analyses of myopathy were performed for these five DDI pairs. In the first myopathy analysis, the total number of medications ordered during the drug exposure window was added as a covariate in the logistic regression. This variable was used as a surrogate marker for the comorbidities of a patient. The average number of medications used by individuals during the drug exposure window was 3.6 with SD = 2.4. Table 4 presents the five DDI effects on myopathy after adjusting for the total number of medications. Compared to table 3, all the single drug myopathy risks and drug combination risks were reduced after justifying for the number of co-medications. The DDI evidence became even more significant (p-values less than 3e-12), and risk ratios became even bigger, between 2.72 and 7.00. The medication frequency itself was also associated with increased myopthay risk. The addition of one co-medication was associated with an increased myopathy risk between 0.6% and 0.9% in testing the 5 DDI pairs. All p-values are less than 2e-16.

**Table 4:** DDI-Myopathy analysis adjusted for age and sex and co-medications.

drug 1	drug 2	Enzymes	Risk1	Risk2	Risk12	Risk Ratio	p-value
**Loratadine**	**Simvastatin**	CYP3A4	0.0085	0.0016	0.027	2.72	2.95E-12
**Loratadine**	**Alprazolam**	CYP3A4	0.0086	0.0041	0.045	3.58	<2.00E-16
**Loratadine**	**Duloxetine**	CYP2D6	0.0084	0.019	0.080	2.89	<2.00E-16
**loratadine**	**ropinirole**	CYP2D6	0.0083	0.0028	0.078	7.00	<2.00E-16
**promethazine**	**tegaserod**	CYP2D6	0.0040	0.013	0.089	5.10	<2.00E-16

Note: Risk1 and risk2 are myopathy risks for drug 1 and drug 2 respectively. The risk-ratio is calculated as risk12/(risk1+risk2). The p-value is calculated from a multivariate logistic regression, in which age, sex, and co-medications were included.

In the second myopathy analysis, only the first myopathy events were considered, because co-medications administered after the first myopathy event but before the follow-up myopathy events were potential confounders. In other words, it was difficult to justify whether the co-medication drug exposure resulted from the myopathy or caused myopathy. Table S3 presents the data analysis for the DDI pairs: (loratadine, simvastatin), (loratadine, alprazolam), (loratadine, ropinirole), (loratadine, duloxetine), and (promethazine, tegaserod). Their relative risks are (1.34, 1.38, 1.38, 1.81, 1.70) respectively, the p-values are (3.20E-03, 2.1E-05, 9.4E-04, 3.1E-03, 2.3E-03) respectively. This analysis based on first myopathy event with these five selected DDI pairs confirmed the trend of our previous synergistic DDI analysis.

## Discussion

### DDI Text Mining and DDI Prediction

Unlike DDI signal detection from AERS by Dr. Altman's group [10], we enriched our EMR signal detection by focusing on CYP-mediated DDIs that were mined and predicted from PubMed abstracts. There are multiple recent publications on drug interaction text mining. Two automatic literature mining systems were developed to predict drug interactions based on their associated metabolism enzymes [13], [14]. An evidential approach was developed to differentiate *in vitro* and *in vivo* DDI studies, curate drug metabolism and inhibition enzymes, and predict DDIs based on their pertinent enzymes [15]. Our text mining approach took advantage of these two methods, i.e. metabolism based DDI prediction; and emphasized the text mining performance more stringently. The IR step of our method is an automatic algorithm, which has high recall rate (0.97); while the IE step is a manual curation step, with high precision (100%). In addition, we implemented CYP enzyme probe substrates and inhibitors from the FDA guidance into the literature mining method. This strategy supplies information on the potential mechanism for the predicted DDIs. Our current text mining method focuses on pharmacokinetic-based drug interaction literature, i.e. reported substrate drug exposure changed by drug interaction. Text mining which focuses on pharmacodynamics (PD) DDI literature has been recently discussed [16], [17]. PD DDI literature reports the drug efficacy or side-effect changes, but it usually does not report drug exposure change.

### Lack of Clinical Validation of In Vitro DDIs

Among the 13197 predicted DDIs from in vitro PK study literature mining, 3670 of them may have clinical relevance, i.e. they were taken as co-medications by at least some of the 2.2 million patients in our clinical dataset. However, only 196 of them (5.3%) have been tested in clinical pharmacokinetic DDI trials. Among these 196 clinically tested DDIs, 123 of them (62.7%) showed significant substrate drug exposure increase when co-administrated with the inhibitor. This striking finding calls for further evaluation of those predicted DDIs that have not been subjected to rigorous study. As a matter of fact, all five DDI pairs which showed an increased myopathy risk in our pharmaco-epidemiology study lack clinical pharmacokinetic studies.

### Mechanistic Interpretation of Significant DDIs

**Figure 4:**
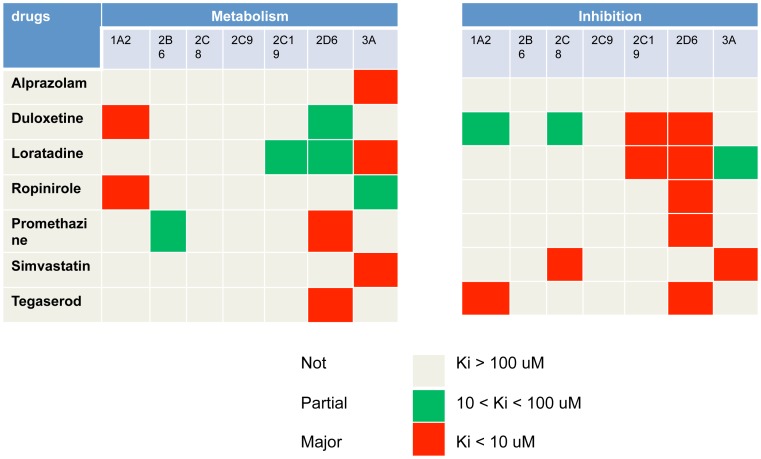
Metabolism enzymes and inhibition potencies of seven drugs. The metabolism enzymes of a drug are characterized with major, partial, or not. The inhibition potencies of a drug are characterized with strong (Ki<10 uM), moderate (10<Ki<100 uM), and weak (Ki>100 uM).

The FDA labels of all 7 of the drugs which comprise the five significant DDI pairs report myopathy related side effects (Table S4). This evidence confirms the myopathy risk for each individual drug. In order to understand the mechanisms of each interaction, we further explored literature regarding those agents. In Figure 4 and Table S5, we integrated information on the metabolism and inhibition enzymes of those 7 drugs from a full-text based literature review of reported *in vitro* studies of the drugs. Table 5 presented the DDI potency prediction for the five DDI pairs. Loratadine (substrate) and simvastatin (inhibitor) were predicted to have a strong DDI through the CYP3A4/5 enzyme. Tegaserod (substrate and inhibitor) and promethazine (substrate and inhibitor) were predicted to have strong DDI through the CYP2D6 enzyme. Their interactions are mixed inhibition and auto-inhibition. The other four drug pairs were predicted to have moderate DDIs: loratadine (inhibitor) and omeprazole (substrate) interact through both the CYP2C19 and CYP3A4/5 enzymes; loratadine (inhibitor) and alprazolam (substrate) interact through CYP3A4/5; loratadine (substrate) and duloxetine (inhibitor) interact through the CYP2D6 enzyme; and loratadine (inhibitor) and ropinirole (substrate) interaction is through CYP3A4/5.

**Table 5:** Predicted DDI potency and CYP enzymes among five DDI pairs.

Drug 1	Drug 2	Enzymes	Metabolism Routes	Inhibition potency	DDI Prediction
**Loratadine**	**Simvastatin**	CYP3A	major	strong	Strong
**Loratadine**	**Alprazolam**	CYP3A	minor	moderate	Moderate
**Loratadine**	**Duloxetine**	CYP2D6	major	moderate	Moderate
**Loratadine**	**Ropinirole**	CYP2D6	major	moderate	Moderate
**promethazine**	**tegaserod**	CYP2D6	minor	strong	Strong

### The Consistency of the Mechanism Interpretation of Two DDI Data Analysis Strategies

Two DDI data analysis strategies were implemented to identify drug-drug interactions associated with an increased risk for myopathy. The first approach employed an additive model coupled with a CYP metabolism pathway enrichment analysis. This strategy stems from the newly formed discovery nature of bioinformatics research, i.e. to search for commonality among many hypothesis tests. The second strategy employed a synergistic model coupled with extensive confounder justification. This strategy follows the more stringent pharmaco-epidemiology considerations, which heavily controls for false positives. Unlike the additive model, the synergistic model can justify the myopathic risk effect from an inhibitor in the presence of other potential confounders. Therefore, the additive model would potentially identify more false positive DDIs. However, the additive model is more powerful than the synergistic model in identifying the true positive DDIs. Many more DDIs were identified by the additive model based DDI analysis than by the synergistic strategy. Because pathway enrichment analysis allows more flexibility toward false positive DDIs, the additive model identified CYP3A4/5 and CYP2D6 enzymes as they have the enriched DDI pairs. Although the synergistic model DDI analysis only inferred five significant DDI pairs, upon additional literature review, it was found that these pairs also showed mechanistic involvement of CYP2D6 and CYP3A4/5 enzymes. The consistency of the mechanistic interpretations of the two separate DDI analysis strategies delivers an encouraging message: the bioinformatics approach and the pharamco-epidemiology approach are complementary and mutually supportive.

### Synergistic DDI Test and DDI Mechanism Based Interpretation

Our synergistic DDI test is a very stringent approach, compared to the additive approach used by the other investigators [9], [18], [19]. We recognize that our synergistic DDI test may exclude some true DDIs. It assumes that all myopathy is the result of drug administration, and patients who don't take the DDI drugs won't have myopathy. However, there is a background rate of myopathy in patients that is not due to either of the two drugs in a specific DDI. If the patients who don't take drugs have a baseline risk of myopathy, the relative risk estimated through our synergistic DDI test will be smaller than the true relative risk. In our follow-up sensitivity analysis, medication frequency was justified in the DDI analysis. This factor would also account for a portion of baseline myopathy risk. Another potential approach to estimate the baseline myopathy risk is to identify a control patient group that matches the demographics, co-morbidity, and co-medication distributions of the group exposed to the DDIs. This approach deserves further investigation.

Like many pharmaco-epidemiology studies using observational data, our analysis of the DDI effect on myopathy has several limitations. Creating an accurate phenotypic definition using billing codes may be unreliable, with both false-positives and false-negatives likely to occur. Our dataset also lacked clinical notes from which more detailed symptom data could be extracted. Further research including validation with manual chart review is necessary to establish optimal phenotypic definitions for myopathy, as well as more granular definitions for myotoxicity and rhabdomyolysis. Further research including validation with manual chart review is necessary to establish optimal phenotypic definitions for myopathy, as well as more granular definitions for myotoxicity and rhabdomyolysis using a combination of ICD9 codes, lab tests, and clinical notes.

Another limitation of our analysis is that it is subject to several potential population bias introduced by the EMR database itself. Our retrospective observational data do not allow for controlling many potential covariates that a traditional prospective study offers. In particular, the race data is not complete in our database. It is also equally challenging to design a prospective study to validate our results from a pharmaco-epidemiology study. Clinical pharmacokinetic studies or further in vitro metabolism/inhibition studies of the selected DDI pairs found to increase myopathy may provide further validation of an interaction between the drugs. We are also looking forward to validating our results in another large EMR database.

Our text mining and DDI prediction is CYP metabolism enzyme based. Therefore, our interpretation of the five significant drug interactions focuses only on CYP drug-drug interaction mechanisms. However, this does not preclude the involvement of other DDI mechanisms, such as drug transporter interactions or pharmacodynamic interactions. In a recent GWAS study, expression of the OATP1B1 transporter was shown to predict myopathy risk associated with simvastatin [20]. Therefore, it is possible that loratadine interacts with simvastatin through this or other transporter mechanisms. Studies are currently underway to further characterize the mechanisms of the five identified DDIs.

### Why Recognized Statin DDIs May Not Be Identified by this Approach

The concomitant use of CYP3A metabolized statins (atorvastatin, lovastatin and simvastatin) with strong CYP3A inhibitiors (e.g. ketoconazole and itraconazole) reportedly increases risk of statin-induced myopathy. In addition, case reports of increased myopathy in transplant recipients being treated with tacrolimus or cyclosporine [21] argue for the avoidance of this combination. The interaction between statins and fibrates, specifically gemfibrozil, leading to increased risk of myopathy is well recognized [22]. Gemfibrozil is a substrate of CYP3A but not a potent inhibitor. Thus, it is likely that this interaction occurs through pharmacodynamic, not pharmacokinetic, based interactions. Although these interactions are widely reported, we found no increased risk of myopathy with concomitant use of ketoconazole, itraconazole, tacrolimus, or gemfibrozil within the CDM database. Their related myopathy risks of these DDIs are reported in Table 6. This finding is likely due to the limitation of our data analysis, in which we define concomitant drug administration by prescription orders that occur within a predefined timeframe. As these drug interactions are well-known, it is likely that although the two drugs may have been ordered within the predetermined time window, the individual may have discontinued one medication before starting the second. For some drugs that are used short-term, e.g. ketoconazole, it will be difficult to identify true concomitant use from prescription records. As a matter of fact, among these statin DDI pairs in Table 6, less than 110 patients took both drugs within the pre-defined one month interval in each pair. This limited our power to detect significant DDIs to less than 15%, if we anticipate a 1.5-fold RR of DDI myopathy. Provided that medication data in our CDM is relatively new, between 2004 and 2009, it is likely that clinicians were aware of potential interactions and thus suggested patients avoid co-administration of these interacting drugs.

**Table 6:** Myopathy relative risk of some statin related drug interaction pairs.

Drug 1	Drug			
	Atorvastatin	Lovastatin	Pravastatin	Simvastatin
**Gemfibrozil**	0.53 (0.22, 1.27); (4113/156140, 614/26961, 6/194)	0.39 (0.16, 1.02); (437/16612, 662/28349, 5/256)	0.38 (0.10, 1.34); (597/20974, 663/28324, 5/278)	0.43 (0.10, 1.76); (10057/445885, 570/24234, 2/100)
**Itraconazole**	0.95 (0.30, 2.96) (4164/157745, 53/2764, 3/69)	0.07 (0.00, 102.7); (442/16833, 56/2825, 0/2)	0.05 (0.00, 24.9); (510/21220, 56/2817, 0/7)	0.26 (0.03, 1.92); (10154/449828, 54/2659, 1/89)
**Ketoconazole**	0.93 (0.66, 1.32) (4130/157280, 424/28661, 32/835)	1.22 (0.46, 3.24); (436/16778, 452/29352, 4/79)	1.63 (0.78, 3.40); (499/21147, 441/29328, 7/111)	0.70 (0.40, 1.21); (10115/448703, 407/27583, 13/499)
**Tacrolimus**	2.25 (0.99, 3.89) (4156/157704, 40/3832, 11/133)	0.23 (0.09, 22.2); (442/16828, 51/3958, 0/9)	0.06 (0.00, 29.6); (510/21225, 51/3957, 0/7)	0.29 (0.09, 1.05); (10154/449790, 48/3689, 3/286)

Note: The p-values of the synergistic drug interaction tests among these drug pairs are larger than 0.05. In each cell, the reported numbers represent relative risk (95% CI) and (m1/n1, m2/n2, m12/n12), where (n1, n2, n12) are sample sizes for drug exposure groups of drug 1 alone, drug 2 alone, and both drugs, respectively; and (m1, m2, m12) are myopathy frequencies for drug exposure groups of drug 1 alone, drug 2 alone, and both drugs, respectively.

### A Combination of Literature Based Discovery and Electronic Medical Record Assessment Is a Powerful Translational Bioinformatics Approach in Predicting Metabolism Based DDIs and Evaluating Their Clinical Significance

As described in the introduction, an *in vitro*, an *in vivo*, or an *in populo* pharmacologic study alone cannot cover the whole spectrum of mechanistic and clinically significant DDI research. These studies usually focus on a few drug pairs for one or a limited number of metabolizing enzymes or transporters at a time. In this paper, we combined a literature discovery approach and a large EMR database validation method for novel DDI prediction and clinical significance assessment. The scale of our research covered all FDA approved drugs. The literature based discovery approach predicted new DDIs and their associated CYP-mediated metabolism enzymes. The clinical significance of these interactions was then assessed in large database of electronic medical records. This translational bioinformatics approach successfully identified five DDI pairs associated with increased myopathy risk. Compared to traditional *in vitro*, *in vivo*, and *in populo* DDI studies, our proposed translational bioinformatics approach covers a broader spectrum and identifies risk on a larger scale. It certainly motivates more *in vitro* studies to investigate alternative DDI mechanisms and more clinical pharmacokinetics study to investigate the clinical significance of these DDIs.

## Methods

### INPC CDM Data Description

The Indiana Network for Patient Care (INPC) is a heath information exchange data repository containing medical records on over 11 million patients throughout the state of Indiana. The Common Data Model (CDM) is a derivation of the INPC containing coded prescription medications, diagnosis, and observation data on 2.2 million patients between 2004 and 2009. The CDM contains over 60 million drug dispensing events, 140 million patient diagnoses, and 360 million clinical observations such as laboratory values. These data have been anonymized and architected specifically for research on adverse drug reactions through collaboration with the Observational Medical Outcomes Partnership project [23].

### Ethics Statement

This CDM model is a de-identified eletronic medical record database. All the research work has IRB approval.

### Candidate Drug Name Preparation for Text Mining

Our drug dictionary consists of 6,837 drugs names that include all brand/generic/drug group names. They were primarily derived from DrugBank [24]. We then excluded non-approved and experimental drugs, and focused only on FDA approved therapeutic agents, which left 1492 unique drug generic names for the mining purpose (Figure 1).

### Mapping between Candidate Drug Names and INPC CDM Medication Data

The INPC CDM data set has 54490 unique drug “Concept IDs”. A Concept ID in the CDM typically maps to an RxNorm clinical drug (e.g., simvastatin 20 mg) or ingredient (simvastatin). Some Concept IDs may contain multiple drug components (e.g., lisinopril/hydrochlorothiazide). Our drug dictionary was mapped to CDM Concept ID's using regular expression matching and manual review. In total, 1293 unique drugs identified from DrugBank were mapped successfully, while 199 drugs could not be matched. The unmatched drugs were categorized as follows: banned drugs, illicit drugs, organic compounds, herbicide/insecticides, functional group derivatives, herbal extract, DrugBank drugs not covered by CDM, and literature only drug names. In our CDM dataset, 817059 patients had medication records available.

### In Vitro CYP Enzyme Substrate and Inhibitor Text Mining and DDI Prediction

**Figure 5:**
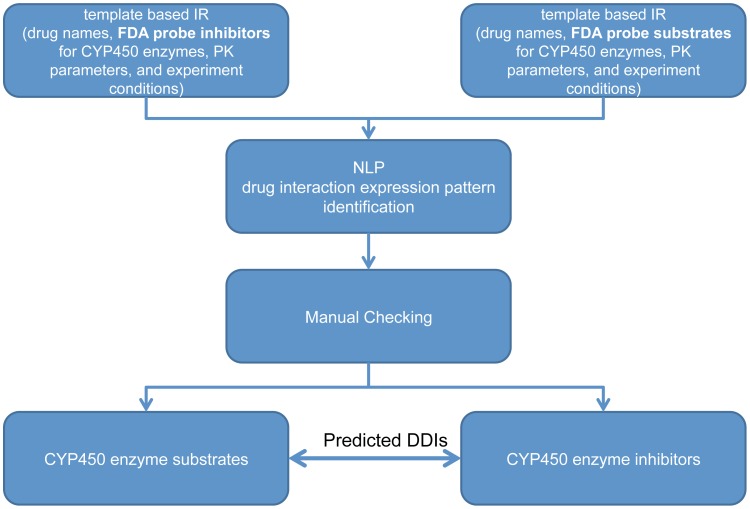
in vitro PK study literature mining flow-chart for CYP substrates and inhibitors, and their DDI predictions.

Literature mining was conducted on 10 CYP enzymes: (CYP1A2, CYP2A6, CYP2B6, CYP2C8, CYP2C9, CYP2C19, CYP2D6, CYP2E1, CYP3A4/CYP3A5) (Figure 5). Please note that these CYPs cover all the major ones, but not all of the CYPs. A probe substrate of enzyme E is defined as being selectively metabolized by enzyme E; while a probe inhibitor of enzyme E selectively inhibits enzyme E's metabolism activity. CYP probe drugs and inhibitors for the DDI text mining approach were selected as those drugs well-established as probes or inhibitors by DDI researchers and defined in the FDA guidance [6]. The in vitro CYP enzyme substrate and inhibitor text mining and the DDI prediction was divided into the following steps.

#### Metabolism enzyme based substrate/inhibitor classification

A drug's ability to be metabolized or inhibited by a specific CYP enzyme is categorized by its published enzyme-based *in vitro* experiments. If drug A was shown to have reduced metabolism by enzyme E with enzyme E's probe inhibitors in an *in vitro* experiment, drug A is enzyme E's substrate. If drug B was shown to inhibit enzyme E's metabolic activity toward enzyme E's probe substrates in an *in vitro* experiment, drug B is enzyme E's inhibitor.

##### *Information Retrieval (IR)*

Information Retrieval (IR) step is a two-step rule based approach. In step one, a template (comprising key terms) was constructed to retrieve PubMed abstracts. The template included required terms: targeted drug name, targeted enzyme name, enzyme specific probe substrates or inhibitors, experiment key terms (i.e. cell systems and equipment set-up), and experiment type (experiment design and parameters); and it included prohibited terms, mostly related to cancer studies. In step two, a natural language processing (NLP) based filter was developed to check the expression patterns in each sentence and decide whether an abstract has DDI-relevant sentences.

In describing the IR process, we will reference the following symbols: O1 denotes inhibitor/inhibit; O2 denotes substrate, probe, metabolized by, or catalyze; O3 denotes inducer/induce; INT denotes interaction, interference, affect, and impact; D denotes drug; and E denotes enzyme. Using these symbols, the patterns are defined as [DEO] : D <D, D…> <not> E O : “drug is (not) enzyme's substrate”; [DOE] : D <not> O E : “drug inhibits enzyme”, “drug is an inhibitor of enzyme”; [EOD] : E <not> O1 <O3> by D : “enzyme is induced by drug”; [IDD1] : <not> INT between D and D : “there is not interaction between drug A and B”; [IDD2] : <not, no> INT D on D : “no impact of drug A on B”; [DID] : D <not, no> INT D : “drug A does not interact with drug B”; Note : also add [OED, ODE, EDO]. Using these expression patterns, a search algorithm was developed to scan each sentence of an abstract, scan for the existence of these patterns, and output the sentence and any DDI patterns/instances.

##### *Information Extraction (IE)*

The Information Extraction (IE) step was conducted manually using following key criteria: 1) only consider human liver hepatocytes/microsomes or recombinantly expressed CYP enzyme systems; 2) only consider FDA probe substrates and inhibitors to determine a drug's metabolism or inhibition potential among the 10 CYP enzymes; 3) only consider drug synonyms covered by our dictionary. In total three curators worked together to perform this manual IE step. First, two curators independently went through all the abstracts from the IR step. The non-overlapped abstracts and a random subset of 20% of the overlapped abstracts were then independently validated by the third curator. This curation plus validation step ensures a high degree of precision in the information extraction process.

##### *Recall rate estimation for the IR steps*

All of the abstracts identified from the IR step (true positives) were combined with a random subset of PubMed abstracts (n = 10,000) (false positives), where the overlapped ones were true positives. The recall rate was calculated as the percentage of true positive abstracts been selected by the IR algorithm.

##### *DDI prediction*

Enzyme E's substrates and inhibitors that were mined from the literature were paired to establish the predicted enzyme E DDIs. At this point, the DDI prediction is based only on the text mining results.

##### *DDI potency prediction*

Each drug's metabolism enzyme information was further reviewed in the full text papers and the extent of metabolism by each enzyme was categorized as one of three groups: major, minor, or not involved. The inhibition enzyme information for each drug was also categorized as one of three groups: strong, moderate, or not involved); and they are based on numerical values of K_i_: <10 uM, 10–100 uM, or >100 uM, respectively. A DDI is concluded as a strong DDI pertinent to enzyme E, if enzyme E is the major metabolism route for at least one drug of the drug pair, and if the other drug shows strong inhibition potency of enzyme E.

### In Vivo DDI Text Mining

*In vivo* DDI text mining was conducted on those predicted DDI pairs from *in vitro* DDI text mining (Figure s1). It is broken down the following steps.

#### In vivo DDI definition

If drug A was shown to have increased systemic exposure by the co-administration of drug B, then A and B have pharmacokinetics drug interaction. The increased systemic drug exposure is usually measured by the area under the drug concentration curve ratio (AUCR), half-life ratio, Cmax ratio, metabolic ratio, or steady state drug concentration ratio.

#### Information Retrieval (IR)

The IR step was again a rule-based approach. A template (key terms) was constructed to retrieve PubMed abstracts. The template included required terms: targeted drug names, clinical trial design, route of drug administration, and PK parameters. The prohibited terms included animal names and *in vitro* terms.

##### *Information Extraction (IE)*

The IE step was again conducted manually as follows: We checked p-values for an increased AUC, Cmax, half-life, or steady state concentration in a drug interaction study. If p<0.05, a DDI was concluded. If there was no p-value reported, we evaluated the change in pharmacokinetic parameter (Cmax, AUCR, half-life, etc). If the fold-change of the parameter of interest was larger than 2.0, a DDI was concluded. Studies in pregnant women and newborns were excluded. We only considered drug synonyms covered by our dictionary. Three curators worked together in this manual IE step. Two curators independently went through all the abstracts from the IR step. A random subset of 20% of the overlapped abstracts and those which were not agreed upon by the initial two curators, were independently validated by the third curator.

##### *Recall rate estimation for the IR step*

All of the abstracts identified with from the IE step (true positives) were combined with a random subset of PubMed list (n = 10,000) (false positives), where the overlapped ones were true positives. The subset was subjected to our proposed IR step, and the recall rate was calculated as the percentage of true positive abstracts selected by the IR algorithm.

#### Myopathy Definition

Our health outcome of the interest (HOI) for this task is *myopathy*, which has a number of potential clinical manifestations [22]. This phenotype is mapped to the CDM condition concept ids (Table S6), as the primary myopathy phenotype in our data analysis. In our CDM dataset, 74584 patients had at least one myopathy symptom between 2004 and 2009.

### Pharmacoepidemiology Study Design of Drug Interactions and Myopathy

#### Retrospective cohort study

**Figure 6:**
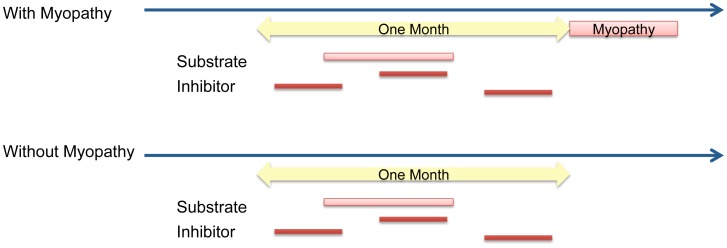
Pharmaco-epidemiology design for myopathy cases and controls in the electronic medical records.

Among patients having a myopathy event, the drug-condition relationship is anchored by the date of myopathy. Any drug exposure occurring within a one month window before the diagnosis of myopathy is considered a positive exposure. If a substrate falls within this window but no inhibitor is present, the event is categorized as “substrate alone” exposure; if both a substrate and an inhibitor fall within this window, it is categorized as “substrate+inhibitor” exposure (Figure 6). If a patient does not have a diagnosis of myopathy, the drug exposure period is anchored by the substrate. If there is an overlap between a substrate and an inhibitor within one month (i.e. they are less than one month apart), it will be categorized as the joint exposure; otherwise only substrate exposure is defined (Figure 6). Therefore, the retrospective cohort DDI study is defined by three drug exposure cohorts: substrate alone, inhibitor alone, and substrate/inhibitor combination. In these three cohorts, cases are patients experienced myopathy, and controls are patients who did not.

*Exclusion Criteria:* patients whose first myopathy event were within the first 6 months of the database were excluded, as we cannot rule out additional myopathy events prior to the starting date of database (01/01/2004).

#### Statistical models of the DDI effect on myopathy

**Figure 7:**
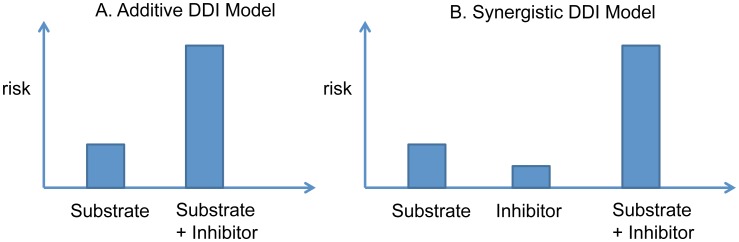
Drug interaction effect models on the myopathy risk. (A) Additive DDI Model; and (B) Synergistic DDI Model.

Because of well-defined cases and controls in this cohort study, a logistic regression model was used to analyze the data. Two logistic regression analyses were performed to test each DDI effect on myopathy. The first is an additive model (Figure 7 A), which tests whether inhibitor plus substrate will lead to an increased myopathic risk comparing to substrate alone. The second statistical model, i.e. synergistic DDI model (Figure 7 B), tests whether the additive myopathic risk from either substrate or inhibitor alone is lower than their combined myopathic risks. In analyzing the DDI synergistic effect, a logistic regression modeled three drug exposure groups: substrate alone, inhibitor alone, and both drugs. The model output their myopathy risk estimates, Risk1, Risk2, and Risk12, respectively. An additional R program was written to calculate their relative risk = Risk12/(Risk1+Risk2), and this statistic was used to test the synergistic effect (i.e., the risk of myopathy for those taking both medications compared with taking each medication individually).

The additive model cannot differentiate whether the increased myopathic risk is inherent to the inhibitor or if it is the effect of a drug interaction leading to increased substrate drug exposure. The synergistic model can identify a greater than expected additive risk of myopathy from the two drugs, indicating a drug-drug interaction. On the other hand, the synergistic model is less powerful in identifying the true DDI than the additive model.

#### Hypothesis testing, hypothesis generation and false positive control

Our primary goal was to identify clinical DDIs resulting in increased risk of myopathy based on the CYP-mediated DDI's identified form literature abstract data. Our hypothesis was that individuals treated with the combination of interacting drugs would have increased risk of myopathy compared to individuals treated with either drug alone (additive model). These hypotheses were tested in the EMR data set, and Bonferroni justification was implemented for the family wise type I error. DDI was also tested among any drug combination effect on myopathy, and these tests serve as the hypothesis generation, instead of the hypothesis testing. In addition, statistical enrichment analysis is performed to identify over-represented CYP enzymes comparing to the rest of the enzymes [25].

### Confounder Considerations

Demographic variables, age and sex, were justified in the DDI association analyses. The total number of different medications ordered during the one month drug exposure window was used as a covariate in the logistic regression. It serves as a surrogate of the patients' overall health status, and justifies for myopathy effects from medications other than the hypothesized DDI drug pair. It is recognized that an individual patient can experience multiple myopathy events. Our drug-condition model considered two situations: all myopathy events and the first myopathy event. The advantage of selecting the first myopathy event is that it is not confounded with other medications taken between the first and the follow-up myopathy events. However, limiting the data to first myopathy even reduces the sample size, and thus the power to identify a DDI. DDI pairs, in which at least one drug was prescribed to treat symptoms of myopathy (e.g. narcotic and non-steroidal analgesics), were excluded from the DDI tests. However, the patients prescribed these drugs are kept in the data analysis.

## Supporting Information

Figure S1 *In vitro* DDI literature mining flow chart.
(TIF)

Table S1 CYP pathway based categorizations of text mined drug from published *in vitro* studies.
(TIF)

Table S2 CYP pathway enrichment analysis of DDI associations of the myopathy risk.
(TIF)

Table S3 Significant synergistic DDI effects on the myopathy risk. Only the first drug exposure/myopathy event was counted for each subject. Risk1 and risk2 are myopathy risks for drug 1 and drug 2 respectively. The risk-ratio is calculated as risk12/(risk1+risk2). The p-value is calculated from a multivariate logistic regression, in which age and sex were included.
(GIF)

Table S4 Myopathy related adverse drug reactions from FDA labels.
(TIF)

Table S5 Literature review on drug metabolism and inhibition of the seven drugs. We included both *in vitro* and *in vivo* DDI studies.
(XLSX)

Table S6 Myopathy Concept IDs in the Common Data Model.
(XLS)
