# Supplementary figures and images for: Literature Based Drug Interaction Prediction with Clinical Assessment Using Electronic Medical Records: Novel Myopathy Associated Drug Interactions
Source: PLoS Comput Biol. 2012 Aug 9;8(8):e1002614. doi: 10.1371/journal.pcbi.1002614 (PMC3415435; doi:10.1371/journal.pcbi.1002614)

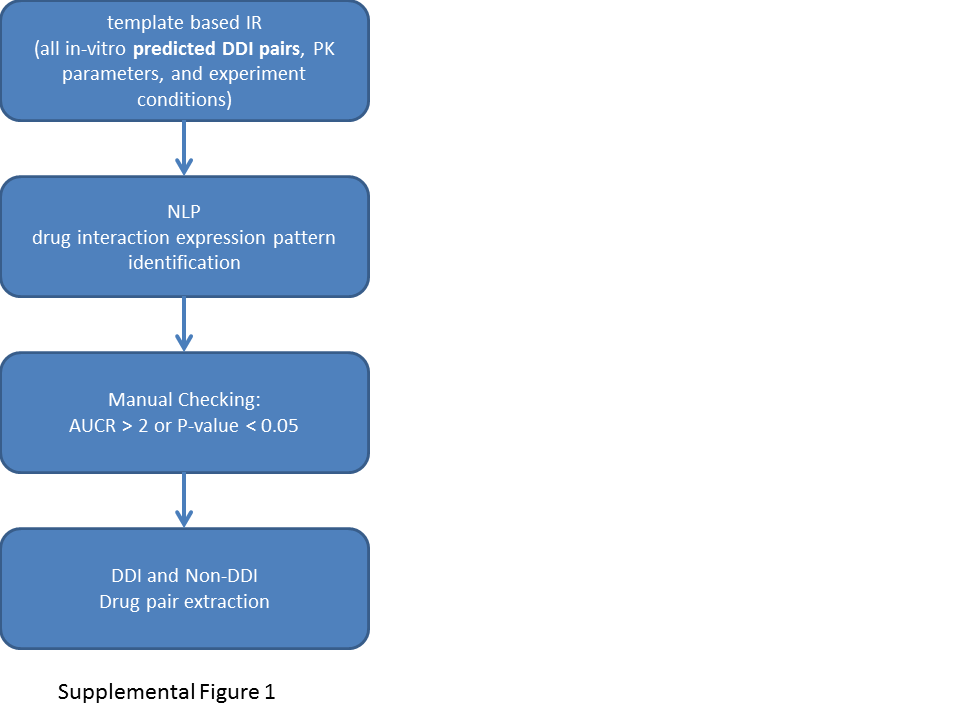

Supplement: Figure S1 — In vitro DDI literature mining flow chart. (TIF) [file pcbi.1002614.s001.tif]

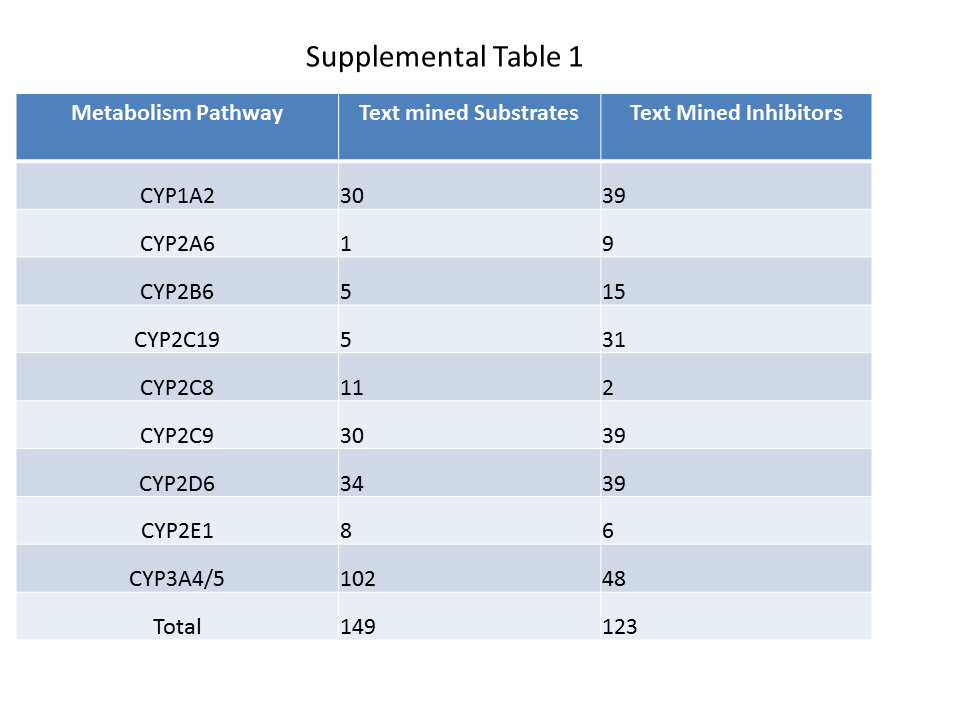

Supplement: Table S1 — CYP pathway based categorizations of text mined drug from published in vitro studies. (TIF) [file pcbi.1002614.s002.tif]

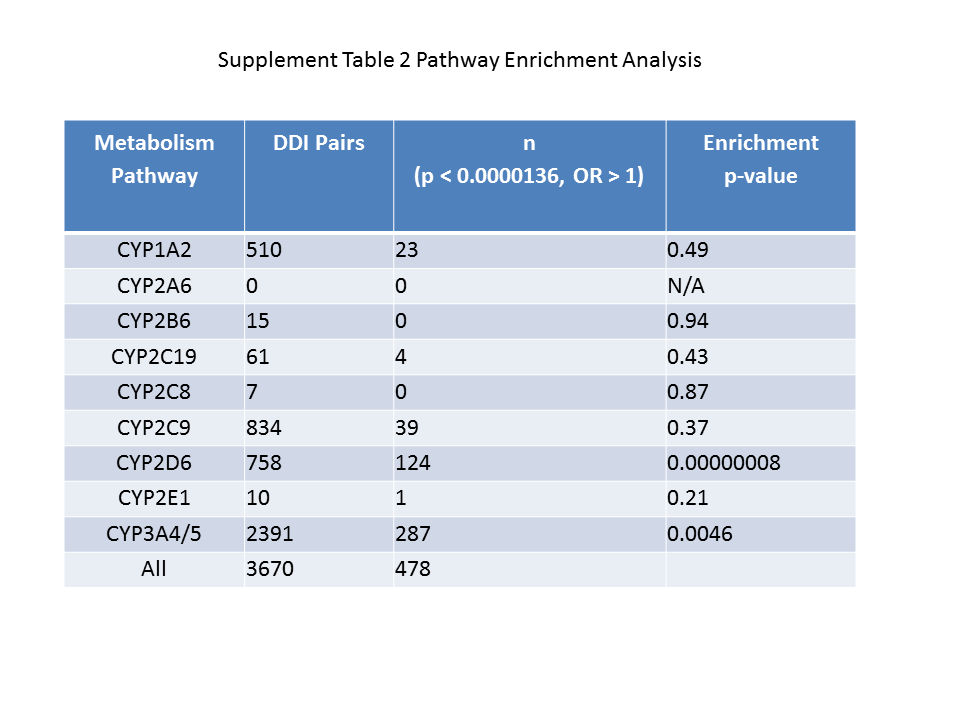

Supplement: Table S2 — CYP pathway enrichment analysis of DDI associations of the myopathy risk. (TIF) [file pcbi.1002614.s003.tif]

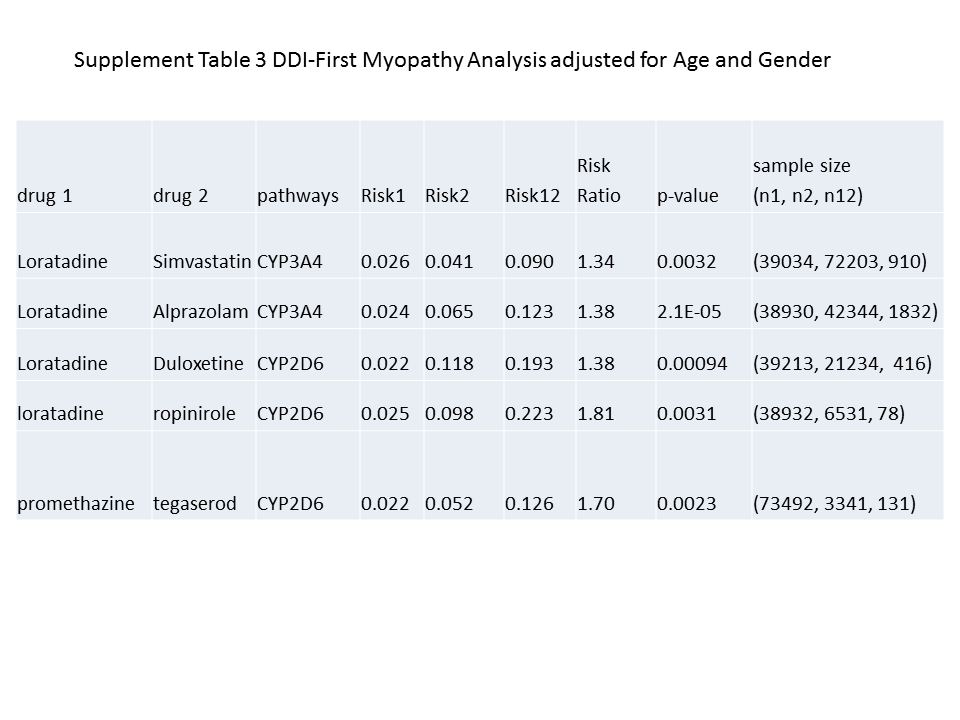

Supplement: Table S3 — Significant synergistic DDI effects on the myopathy risk. Only the first drug exposure/myopathy event was counted for each subject. Risk1 and risk2 are myopathy risks for drug 1 and drug 2 respectively. The risk-ratio is calculated as risk12/(risk1+risk2). The p-value is calculated from a multivariate logistic regression, in which age and sex were included. (GIF) [file pcbi.1002614.s004.gif]

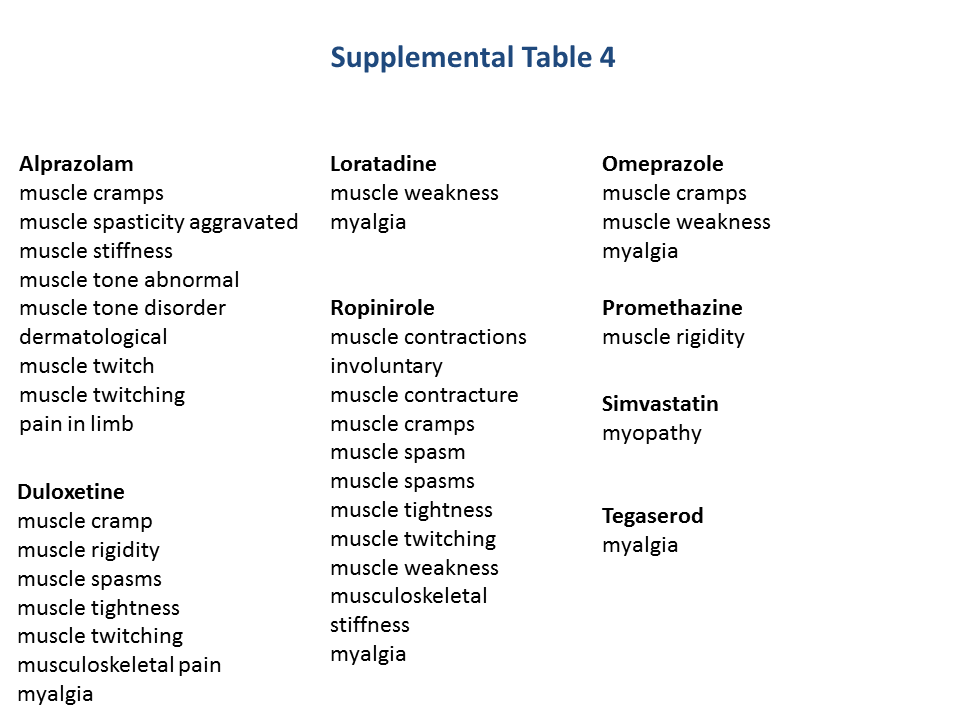

Supplement: Table S4 — Myopathy related adverse drug reactions from FDA labels. (TIF) [file pcbi.1002614.s005.tif]
